# Supplementary material for: Comparison of the selection of nasotracheal tube diameter based on the patient’s sex or size of the nasal airway: A prospective observational study
Source: PLoS One. 2021 Mar 8;16(3):e0248296. doi: 10.1371/journal.pone.0248296 (PMC7939375; doi:10.1371/journal.pone.0248296)
Supplement: S1 Text — (DOCX) [file pone.0248296.s003.docx]

S1 Text. Study details

1. Study approval and patient enrollment

The study protocol was approved by the institutional review board of Severance Hospital, Yonsei University Health System, Seoul, Korea (IRB no. 1-2017-0048, September 6, 2017) and registered at ClinicalTrials.gov (NCT03282604; data collection started on September 25, 2017, and ended on December 6, 2018). From September 2017 to December 2018, we enrolled patients aged 20 to 70 years (American Society of Anesthesiologists, class I–II) who were scheduled for nasotracheal intubation. We excluded patients who were pregnant, had difficulty breathing, had impaired cervical motion, had undergone emergent surgery, had a nasal disease, or refused to participate in the study

1. Anesthetic management and measurement

After entering the operating room and lying down in the supine position, the patients were asked to block each nostril in turn and identify the one that allowed more comfortable breathing. Standard monitors for pulse oximetry, 3-lead electrocardiography, and non-invasive blood pressure measurement were attached. Anesthesia was induced using 1–2 mg kg^-1^ propofol (Fresofol 1% MCT; Fresenius Kabi Austria GmbH, Graz, Austria), 0.5–1.0 µg kg^-1^ remifentanil (Ultian; Hanlim Pharm. Co., Ltd., Seoul, Korea), and 0.6 mg kg^-1^ rocuronium (Rocumeron; Ilsung Pharmaceuticals Co., Ltd., Seoul, Korea). Mask ventilation was performed using oxygen at 5 L min^-1^ and sevoflurane 4.0 vol %. Complete muscle relaxation was assessed at the adductor pollicis muscle by a supramaximal train-of-four stimulus applied to the ulnar nerve using a peripheral nerve stimulator (Innervator 252; Fisher & Paykel Healthcare, Auckland, New Zealand).

1. Measurement of maximum allowable tube diameter

After ensuring complete muscle relaxation, the mask ventilation was stopped and nasopharyngeal airways (PVC airway; SunMed, Grand Rapids, MI, USA) with internal diameters of 6.0, 6.5, 7.0, 7.5, and 8.0 mm, well lubricated with jelly, were inserted through the patient's preferred nostril in the order of increasing size of the airways. The resistance encountered by the nasopharyngeal passage during insertion of the nasopharyngeal airways was recorded as mild or moderate. Smooth insertion with little or slight friction was graded as mild resistance, and obvious resistance with potential risk of intranasal abrasion was graded as moderate resistance. If moderate resistance was felt, the attempt to insert the nasopharyngeal airway was stopped and the airway removed from the patient’s nose. The airway’s fit relative to the nasopharyngeal passage was assessed based on this resistance. The size of the airway was considered to be correct when mild resistance was felt, and this size was considered as the maximum allowable tube diameter. The airway was considered to be oversized when moderate resistance was felt.

1. Measurement of airway length (from the nostril to vocal cord, and carina)

Mask ventilation was resumed for 1 minute to maintain oxygen levels, and a flexible fiberoptic bronchoscope (Olympus LF-GF; Olympus Optical Co., Tokyo, Japan) was inserted into the nasal cavity.

When the bronchoscope approached the carina, adhesive plasters were applied to the bronchoscope at the nostril. After the bronchoscope was gradually withdrawn until its tip reached the vocal cords, a second adhesive plaster was applied to the bronchoscope at the nostril. We then removed the bronchoscope from the patient’s airway and measured the length between the two adhesive tapes using a ruler; this was the length marked as the distance between the vocal cords and carina. During this measurement, mask ventilation was resumed and nasotracheal intubation was performed using Ivory PVC Portex North Facing Nasal Soft-Seal Cuffed Polar Preformed Endotracheal Tube (Smiths Medical International, Hythe, United Kingdom). The ideal tube position was defined as the position in which the tube’s cuff or tip was within the safety margin, with the cuff below the vocal cords or tip above the carina even in the various positions that are induced by possible head and neck movements. With the neck in a neutral position, we measured the distance between the tube’s cuff and vocal cord to be less than 20 mm, and the distance between the tube’s tip and carina to be less than 30 mm. This indicated a risk of damage to the vocal cords, accidental extubation, or endobronchial intubation since the tube can be moved outside the safety margin after the patient’s neck position is changed. Using these criteria, the maximum allowable distance between the tube’s cuff and tip was calculated based on the measured distance between the vocal cords and carina.

1. Statistical analysis

Clinically significant factors among the preoperative characteristics, including age, sex, height, and weight were entered into a multivariable logistic regression model to assess their impact on the maximum allowable distance between the tube’s cuff and tip through a stepwise variable selection. We performed a multivariable ordinal logistic regression analysis for identifying predictors of the maximum allowable tube diameter. After identifying the appropriate predictors to select the tube diameter, we aimed to measure the difference in safety margin between the maximum allowable distance between the tube’s cuff and tip, and the distance between the selected tube’s cuff and tip according to the identified significant preoperative factors. The number of patients who had an appropriate distance between the tube’s cuff and tip within the maximum allowable distance of the patient’s airway was also analyzed and compared for two selection methods of tube diameter.
